# Dental Education.—Twenty-Sixth Annual Session Ohio College of Dental Surgery

**Published:** 1871-10

**Authors:** 


### DENTAL EDUCATION.—TWENTY-SIXTH ANNUAL SESSION OHIO COLLEGE OF DENTAL SURGERY

The twenty-sixth annual session of the Ohio College of Dental Surgery was commenced yesterday in the College Building, on College street, which has been thoroughly renovated and repaired.

The session opens with a large class under auspices of the most flattering character.

Professor David Vaughan, whose name is prominent as well as honored among the scientific men of the day, has accepted the chair of chemistry, and added his magnificent apparatus to the facilities of the institution.

This is the first professional institution in the country that adopted the system of classified teaching, which is now in operation five years. The medical department of Harvard College adopted it this year, and proclaimed it as a novelty. The Miami Medical College of Cincinnati has had it in operation three years.

The annual announcement in speaking of this plan, says:

"The pupils will be divided as heretofore into senior and junior classes; the admission to the one being gained only by the mastery of the studies of the other. The present curriculum of Dental study including Anatomy, Physiology, Histology, Pathology, Materia Medica, Therapeutics, Organic and Inorganic Chemistry, Metallurgy, Operative Dentistry, Hygiene, Microscopy and Mechanical Dentistry, presents altogether too wide a range of thought, and too many studies to be pursued with profit at once. So manifest are the advantages accruing from the classification introduced by this institution, imperfect though it be, that no one in anywise familiar with it would regard it for a moment as advantageous or desirable in any respect to make even the slightest retrograde movement, or to return to the practice of teaching all alike, regardless of their relative attainments.

"The lectures are arranged to meet the wants of students, and will be as numerous as can be heard with profit.

"The lectures and clinical instructions in the Infirmary and Laboratory will fully occupy the students' time, and they should be ready to give them their undivided attention from the first day of the session."

Microscopy is taught in this college under a competent, practical instructor, provided with the best instruments and microscopical apparatus in the West.

This college also affords rare opportunities to the students in operative dentistry. All the principles in detail involved in its practice are presented to the students, who are, in fact, required to thoroughly master them, as a basis for treatment and manipulations.

A Dental Infirmary is connected with the college, where every member of the senior class is required to spend a portion of each day in the work of the Infirmary.

The Faculty are:

James Taylor, M. D., D. D. S., Emeritus Professor of Institutes of Dental Science.

C. Kearns, M. D., Professor of Anatomy.

Edward Rives, M. D., Professor of Histology, Physiology and Pathology.

D. Vaughan, Professor of Chemistry, Materia Medica and Therapeutics.

F. Brunning, M. D., Professor of Pathology.

J. Taft, D. D. S., Professor of Operative Dentistry, Dental Hygiene and Microscopy.

C. M. Wright, D. D. S., Professor of Mechanical Dentistry and Metallurgy.

Wm. Taft, D. D. S., Professor of Clinical Dentistry.

A. Schwagmeyer, M. D., Demonstrator of Anatomy.

The regulations of the college are:

"1. An extension of the session to five months.

"2. A preliminary examination, the requirements of which shall be a good English education.

"3. There shall be two classes, junior and senior; the first shall consist of first course students, the second of those who are candidates for graduation.

"4. The studies of these classes shall be arranged as follows:

"First Year or Junior Class—Anatomy, embracing Dissections, Physiology, Histology, Inorganic Chemistry, Metallurgy, and Mechanical Dentistry.

"Second Year or Senior Class—Histology, Pathology, Dissections, Organic Chemistry, Therapeutics, Operative Dentistry, and Dental Hygiene.

"5. Members of the Junior Class will be required to pass an examination on the branches studied before entering the Senior Class. This may be at the close of the Junior or the beginning of the Senior course, at the option of the student. When this examination is satisfactory, a certificate of the fact, bearing the seal of the College, shall be given to the student, which shall entitle him to enter the Senior Class.

"6. Applicants for admission to the Senior Class must pass a satisfactory examination on the Junior course, except when, in special cases, the Faculty may allow them to take a part of the Junior Course in connection with the Senior, in which case this part of their examination will be deferred till the close of the Senior term."—*Cincinnati Commercial.*

